# The Clinical Efficiency of Positive Airway Pressure Treatment

**DOI:** 10.1155/2013/245476

**Published:** 2013-04-29

**Authors:** Sabri Koseoglu, Aykut Ikinciogullari, Mehmet Ali Cetin, Gokce Saygi Uysal, Rauf Oguzhan Kum, Berna Arli

**Affiliations:** ^1^Ankara Numune Education and Research Hospital, ENT Clinic, 06640 Ankara, Turkey; ^2^Kulu State Hospital, ENT Clinic, Konya, Turkey; ^3^Ankara Numune Education and Research Hospital, Neurology Clinic, 06640 Ankara, Turkey

## Abstract

*Objectives*. The aim of this study was to evaluate the clinical efficiency and compliance of positive airway pressure (PAP) treatment. *Materials and Methods*. This study was conducted on moderate-severe obstructive sleep apnea syndrome (OSAS) patients who admitted to Ankara Numune Hospital Sleep Center between 2008 and 2012. Seventy-five patients with moderate-severe OSAS who were using PAP treatment regularly were enrolled in the study. Patient's usage data, Epworth sleepiness scale (ESS) scores, and the differences in complaints of OSAS were recorded. *Results*. The overall complaints were improved when compared to pretreatment period. Particularly there was improvement in apnea, snoring, excessive daytime sleepiness, fatigue, and sleep quality. *Conclusion*. PAP is effective in reducing symptoms in people with moderate and severe OSAS. To inform the patients with details and the creation of strategies for close followup are necessary for improving the compliance of the patients. Trial registration number: ACTRN12613000373774.

## 1. Introduction

Positive airway pressure (PAP) is the first line treatment of moderate and severe obstructive sleep apnea syndrome (OSAS). Disturbance of sleep quality, excessive daytime sleepiness, apnea, deterioration of life quality, and also the presence of cardiovascular, metabolic, neuropsychiatric, and urogenital disorders such as hypertension, diabetes mellitus, dislipidemia, metabolic syndrome, panic attack, depression, stroke, and erectil dysfunction show the importance of the treatment [1–7]. Previous studies showed that PAP treatment improves the symptoms of OSAS, rate of comorbid disorders, cardiovascular morbidity, and mortality. The positive consequences of PAP treatment result higher compliance of patients [1–6, 8].

PAP compliance is the ratio of the patients that use the treatment regularly to the total number. Compliance problem is frequently encountered in OSAS patients. Some publications notified that 29–48.2% of the patients do not use the PAP treatment [8, 9]. The adverse effects such as difficulty to exhale against positive pressure, skin reactions, unfit or very tight mask, nasal blockage, dryness of throat, and aerophagia are among the factors that affect the compliance negatively [10].

The aim of this study was to evaluate the knowledge of patients about the usage of device, problems encountered during the usage of device, Epworth sleepiness scale (ESS), comparison of the complaints of the patients before and after the usage of device, and the alterations of nocturia by questionnaires. 

## 2. Materials and Methods

This study was conducted on moderate-severe OSAS patients who admitted to Ankara Numune Education and Research Hospital Sleep Center between 2008 and 2012. Patients with apnea hypopnea index (AHI) above 15 measured by all night polysomnographic studies (PSG) were enrolled to the study. PSG was performed during patients' spontaneous sleep with the supervision of a technician. Electroencephalogram (EEG), electromyogram (EMG-submental and left-right tibialis anterior), electrooculogram (EOG, left-right), nasal airflow, thoracic and abdominal respiratory effort, blood oxygen saturation (by pulse oximetry), and body positions were recorded all night long. These data were also scored manually by using Alice PSG system by an ENT physician with a sleep certificate.

Moderate-severe OSAS patients with AHI >15 were informed in detail about the risk of their disease, possible morbidity and mortality, and the importance of treatment before titration. This information was given by the experienced ENT physicians who performed the PSG scoring. 

Among these, 153 of patients performed automatic positive airway pressure titration all night long. Titration data was scored manually. Automatic positive airway pressure (APAP) device was given to supine-dependent and/or rapid-eye-movement- (REM-) dependent patients, and bilevel positive airway pressure (BiPAP) device was given to patients with pressure above 12 cm H_2_O in automatic PAP titration, chronic obstructive lung disease, and/or obesity hypoventilation syndrome, and (continuous positive airway pressure) CPAP was given to other patients.

As 35 of the patients could not be reached, they were excluded from the study. Total 118 patients were reached, and informed consent were taken.

43 patients (43/118, 36.5%) did not use PAP treatment. 75 patients (75/118, 63.5%) kept going on with PAP treatment. 

75 patients who were using PAP treatment regularly were enrolled in the study. Patient's usage data, ESS scores, and the differences in complaints of OSAS were recorded (Table 1). 

Control examination of patients was performed, and the routines of device usage and usage time (Table 1), problems encountered during the usage of device (Table 2), Epworth sleepiness scale (ESS), and comparison of the discrepancy of complaints before and after the usage of device were evaluated with questionarries. In Table 3, patients were asked to score the discrepancies of their complaints considering their state prior to treatment with worse (−2), bad (0), good (+1), or better (+2).

## 3. Results

49 (65.3%) of the 75 patients were males and 26 (34.7%) were females. Their mean age was 54.2 ± 6.1 (35–84). 

The mean usage time of device was 15.1 ± 6.03 (6–30) months. Mean orientation time of the patients to the device was detected as 10.5 days (1–90 day). The mean usage time of the device was 6.3 days (4–7). The mean daily usage time was 6.7 (4–10) hours. 

Patients were asked if the device pressure was sufficient or not, and it was revealed that 65 of the patients (86.6%) found it sufficient, 8 (10.6%) of them found more, and 2 (2.6%) of them found less. 

The ratio of the patients and bed partners who were disturbed from the voice of the device was found to be 24/75 (32%).

It was seen that nocturia was reduced in 36 (48%) of the patients, no change in 38 (50.6%) patients, and increased in 1 (1.3%) patient after starting to use the device. 

**Figure 1:**
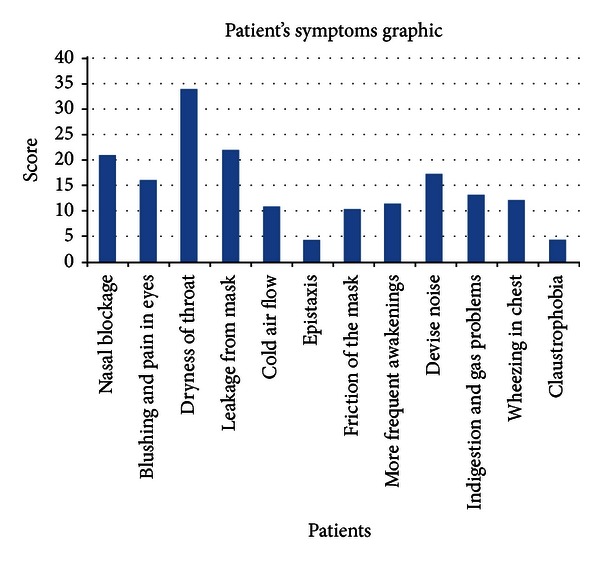
Graphic of patient's complaints.

Problems encountered while using the device were investigated. According to this, most common complaint was dryness of throat (34/75) and then leakage from mask (22/75), nasal blockage (21/75), and excessive noise arising from device (17/75). The problems the patients encountered during the usage of device were summarized in Figure 1.

**Figure 2:**
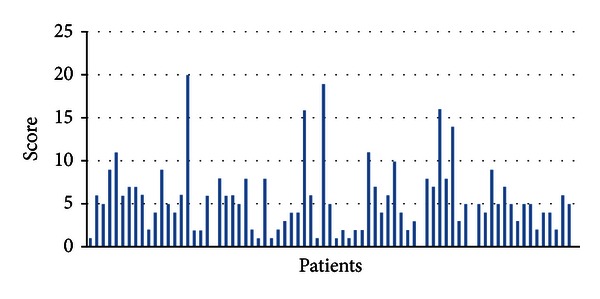
Scoring of Epworth sleepiness scale.

ESS scores of the enrolled patients were shown in Figure 2. When the patients were evaluated according to their mean ESS score, it was found as 5.4. Excessive daytime sleepiness was finished according to ESS. 

**Figure 3:**
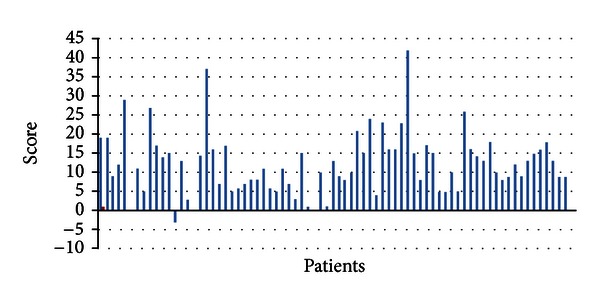
Alterations of OSAS symptoms after PAP treatment by using the scoring in Table 3.

**Figure 4:**
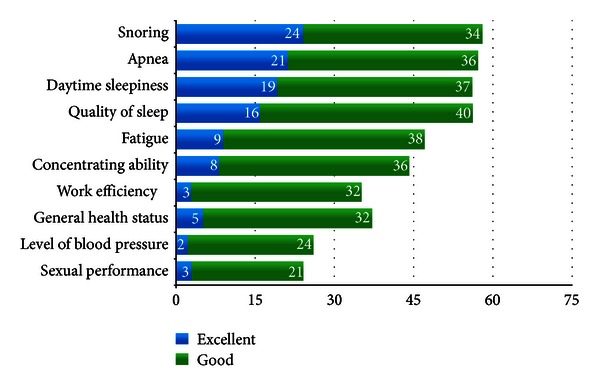
Symptoms which improved after PAP treatment.

Symptoms of patients going on PAP treatment were evaluated before and after the treatment. The results were shown in Figure 3. The overall complaints were improved comparing to pretreatment period. The symptoms with apparent improvement and number of patients were shown in Figure 4. Particularly there was improvement in apnea, snoring, excessive daytime sleepiness, fatigue, and sleep quality. 

## 4. Discussion

PAP treatment is a golden standard therapy method of moderate and severe OSAS [11]. The compliance is low although the results of the treatment are very good. There are variable compliance ratios reported in PAP treatment studies for compliance. In recent studies, it was seen that the compliance increased [12], but in long-term follow-up studies, it was revealed that the compliance decreased [13]. In this study the compliance ratio was found as 63.5% and the mean usage time as 15.1 months. 

The daily usage time among the patients who are going on PAP treatment is reported as 4.7–5.6 h/d [14, 15]. In this study, it was found as 6.7 h/d.

Throat dryness, leakage from mask, rubbing, nasal blockage, and the noise of the device are the most common problems of the patients who are going on treatment [15]. Also, in this study, throat dryness was found to be the most common adverse effect of the treatment. 

It was reported that psychiatric problems such as depression incidence are low in long-term usage [7]. Also, in this study, there was improvement in depression and anxiety in 18 (24%) of the patients. 

It was known that the blood pressure is reduced with CPAP treatment in hypertension and OSAS patients [16]. In this study, it was detected that blood pressure levels were better with PAP treatment in 26 (34.6%) of the patients. 

ESS is a common method to evaluate the excessive daytime sleepiness all around the world. Ozcan et al. revealed that although ESS is not beneficial to determine the excessive daytime sleepiness, the answers of patients can be influenced by the differences between the socioculturel and economic conditions. Therefore, the necessity to create new forms for each country according to their sociocultural and economic conditions was emphasized [17]. Overall, scores below 10 were accepted as normal, above 10 as excessive daytime sleepiness, and above 16 as severe excessive daytime sleepiness [18]. In this study, mean ESS score of the patients was found to be 5.4, so it was concluded that no one has excessive daytime sleepiness. 

It was reported that there was a reduction in chronic fatigue and nocturia and improvement in sexual function and life quality [19, 20]. In this study, there was a reduction in snoring (58, 77.3%), reduction in fatigue (47, 62%), and reduction in nocturia (36, 48%). It was detected that there was an improvement in sexual function (23, 30%) and sleep quality (56, 74.6%). When compared to pretreatment period, significant improvement and recovery were observed in most of the symptoms. 

As a result, PAP treatment has recoverable and tolerable adverse effects. PAP treatment maintain a significant improvement in sleep and life quality. It was revealed that PAP treatment has a significant recovery on the symptoms of OSAS and related diseases. To inform the patients with details and the creation of strategies for close followup are necessary for improving the compliance of the patients. 

## Figures and Tables

**Table 1:** Definition of usage time of PAP device and habits of the patients.

Usage time (month)	
Adaptation time to sleep with the device (day)	
Mean usage time per week (day)	
Mean usage time per day (hour)	
Device pressure (less-more-sufficient)	

**Table 2:** Definition of evaluation of the problems that can be occur during the usage of CPAP.

Complaints	Positive	Negative
Nasal blockage		
Redness and pain of eyes		
Throat dryness		
Leakage from mask		
Cold airstream		
Nose bleeding		
Rubbing of mask		
Wake up more frequently than before		
Excessive noise of device		
Flatulence		
Wheezing		
Claustrophobia		

**Table 3:** Definition of alteration of complaints after PAP treatment.

Complaints	Much worse (−2)	Worse (−1)	No change (0)	Better (+1)	Much better (+2)
Snoring					
Apnea					
Daytime sleepiness					
Quality of sleep					
Fatigue					
Ability of concentration					
Productivity					
Sweeting during sleep					
Sexual performance					
Restless leg					
General health status					
Chest pain at night					
Palpitation					
Blood pressure status					
Regurgitation in supine position					
Amnesia					
Habit of nocturia					
